# Multicomponent exercise program effects on fitness and cognitive function of elderlies with mild cognitive impairment: Involvement of oxidative stress and BDNF

**DOI:** 10.3389/fnagi.2022.950937

**Published:** 2022-08-25

**Authors:** Catarina Alexandra de Melo Rondão, Maria Paula Mota, Maria Manuel Oliveira, Francisco Peixoto, Dulce Esteves

**Affiliations:** ^1^Department of Sports, University of Beira Interior, Covilhã, Portugal; ^2^Camara Municipal do Fundão, Fundão, Portugal; ^3^University of Trás-os Montes e Alto Douro, Vila Real, Portugal; ^4^Research Center in Sports Sciences, Health Sciences and Human Development (CIDESD), Vila Real, Portugal; ^5^Centro de Química, Vila Real, Portugal

**Keywords:** Alzheimer, MCI, dual task, BDNF, oxidative stress

## Abstract

Regular exercise has been shown to be one of the most important lifestyle influences on improving functional performance, and decreasing morbidity and all-cause mortality among older people. However, although there is some evidence on the effects of aerobic training on oxidative stress, there is little information regarding the effects of multicomponent exercise (dual-task training) and combination of exercise with cognitive stimulation on oxidative stress. In this context, the aim of this study was to verify the effects of a multicomponent exercise program on physical fitness and cognitive function in the elderly with mild cognitive impairment and determine the role of oxidative stress and brain-derived neurotrophic factor (BDNF). At baseline, 37 elderly nursing home residents with mild cognitive impairment were divided into two groups: the control group (CG, *n* = 12, 81.8 years) and the experimental group (EG, *n* = 25, 83.2 years). These elderlies followed multicomponent exercise training for 24 weeks, with two sessions per week and 45–50 min per session. The exercises included both aerobic and strength exercises, considering functional movements and light to moderate intensity. Cognitive stimulation comprehended exercises based on word games, puzzles, mathematical calculations, forward and backward counting, computer exercises, exergames, and games on a balanced platform. Physical assessments (weight, height, and body mass index), health and functional parameters (fitness tests: chair stand, arm curls, chair sit-and-reach, eight feet up-and-go, back scratch, 6-min walking, feet together, semi-tandem, and full tandem), lipid profile (total cholesterol, high-density lipoprotein (HDL), low-density lipoprotein (LDL), and triglycerides), measures of lipid peroxidation damage, thiobarbituric acid reactive substances (TBARS), total antioxidant capacity (TAC), and BDNF were measured in plasma, based on which analyses were performed before and after the 24 weeks of the multicomponent exercise intervention. The results showed an overall improvement in physical and functional performance. Regarding biochemical measures, multicomponent exercises lead to a significant decrease in oxidative damage. The results indicate that multicomponent exercise training induces benefits in functional capacity and reduces damage due to oxidative stress.

## Introduction

During aging, atrophy and dysfunction of brain tissue are accompanied by a decrease in learning, memory, and hippocampal neurogenesis that frequently leads to mild cognitive impairment (MCI) (Wang et al., 2016). MCI is defined as a cognitive decline greater than that expected for an individual's age and education level, which does not affect most daily activities but has a high risk of progressing to dementia, particularly Alzheimer's disease (Gauthier et al., 2006). In fact, it has been proposed that the annual progression rates of MCI to dementia range from 10 to 15% (WHO., 2019). There are no curative treatments for dementia or MCI, but it has been estimated that 3% of dementia cases could be prevented by increasing levels of physical activity and exercise for preventing and eventually slowing down the pathological process and dementia-related problems (Sadowsky and Galvin, 2012). In fact, physical exercise has been related to cognitive function(s) through a variety of cellular and molecular processes that induce angiogenesis, neurogenesis, and synaptogenesis, thus improving learning, memory, and brain plasticity (van Praag et al., 1999).

Some of the aforementioned mechanisms that contribute to modulate exercise-induced cognitive improvement are neurotrophins (Radak et al., 2007) and oxidative stress (Cobley et al., 2013). Brain-derived neurotrophic factor (BDNF) is the most abundant neurotrophin, and though it is synthesized in peripheral tissues, such as muscle, liver, adipose tissue, endothelial cells, and immune cells, 75% of its synthesis occurs in different types of brain cells and can be transported outside the brain through the blood–brain barrier (Bathina and Das, 2015). BDNF appears to be essential for neuronal survival during the development and formation of neural networks of the peripheral and central nervous systems and regulates synaptogenesis, synaptic transmission, and plasticity by/through/*via* its tyrosine kinase receptor B (TrkB) (DeLaRosa et al., 2019; Molinari et al., 2020). BDNF and TrkB expression in the hippocampus and temporal cortex decreases over the years in humans, which has been related to the increased problems in learning and memory (Webster et al., 2006). Acute exercise induces an increase in the BDNF levels compared to those produced in the resting conditions, which return to baseline levels within minutes following exercise cessation (Currie et al., 2009). The effects of chronic exercise on BDNF levels have not been consistent, with literature reporting any significant changes (Schulz et al., 2004; Ogonovszky et al., 2005; Schiffer et al., 2009), an increase (Zoladz et al., 2008; Erickson et al., 2011; Voss et al., 2013), or even a decrease in resting values, suggesting that the mode and workload characteristics of the exercise program should be a decisive factor.

Reactive oxygen species (ROS) are highly reactive chemical compounds that are generated during normal metabolic processes, and in excess can damage macromolecules, such as lipids, proteins, and deoxyribonucleic acid (DNA), causing cellular dysfunction and possibly death (Lohr and Browning, 1995). The antioxidant defense system reduces the action of ROS, by preventing, scavenging, and repairing them. Oxidative stress (OS) involves an imbalance between pro-oxidant processes and the antioxidant defense system in favor of the former (Barbosa et al., 2010; Gabriel et al., 2012). An accumulation of oxidized proteins, lipids peroxides, and DNA oxidatively damaged in the brain potentiates neurodegeneration and impairs cognitive function (Radak et al., 2007), which has been demonstrated to be one of the main molecular mechanisms of brain aging and neurodegenerative disorders like Parkinson's disease, Alzheimer's disease, and Huntington's disease (Federico et al., 2012). In the study of *in vitro* animals, it is suggested that oxidative stress, mitochondrial function, and BDNF have a complex and reciprocal relationship. Mitochondrial organelles have a crucial role in adenosine triphosphate (ATP) production through oxidative phosphorylation, a process performed by the electron transport chain (ETC) complexes I through V, and is related to the levels of intra- and extracellular BDNF (Markham et al., 2012; Kim et al., 2015). Moreover, BDNF interacts with ATPase, improving the mitochondrial respiratory coupling (Markham et al., 2004, 2012). Additional studies have shown an inverse relation(ship) between oxidative stress and BDNF levels, indicating that BDNF may play a protective role against oxidative damage in neurons (He and Katusic, 2012; Valvassori et al., 2015), possibly through the increase of the antioxidant capacity of cells (He and Katusic, 2012).

Therefore, physical exercise may mitigate age-related cognitive decline through the modulation of factors participating in the crosstalk between skeletal muscle and the brain, such as neurotrophins and oxidative stress. Several studies described a reduction in the markers of oxidative stress in resting conditions after the implementation of exercise programs (Barbosa et al., 2010; Gabriel et al., 2012).

Regarding the kind of exercise intervention more recommended to this population, recent systematic reviews and meta-analyses (Karssemeijer et al., 2017; Bruderer-Hofstetter et al., 2018; Gheysen et al., 2018; Gavelin et al., 2021) concluded that dual-task or multicomponent exercise is more advantageous than a simple exercise in MCI individuals, since working simultaneously on the physical and cognitive components allows for more stimuli, enhancing neural regeneration by increasing blood flow to the brain, promoting neural growth, maintaining brain function, and improving brain plasticity (Bherer, 2015; Morita et al., 2018). Combining physical and cognitive rehabilitation programs leads to significant improvements in physical fitness and also improves cognitive performance (Karssemeijer et al., 2017; Bruderer-Hofstetter et al., 2018; Gheysen et al., 2018; Zhang et al., 2019; Gavelin et al., 2021).

Considering that aging results in decreased physical and cognitive capacities, particularly more pronounced in frailty populations, and that regular exercise improves the function of most of the organs, can multicomponent exercise prevent or attenuate the decline of physical and cognitive function of elderlies with MCI? Moreover, considering the role of oxidative stress in mediating cell adaptation to exercise, and its relationship with BDNF, what changes are we able to observe in these parameters and how are they related to functional variations in elderlies with MCI? Regarding this, the aim of this study is to analyze the effect of a multicomponent exercise program with stimulation on fitness, cognitive function, plasmatic lipid profile, oxidative stress, and BDNF of elderlies with mild cognitive impairment.

## Methods

### Participants

**Figure 1:**
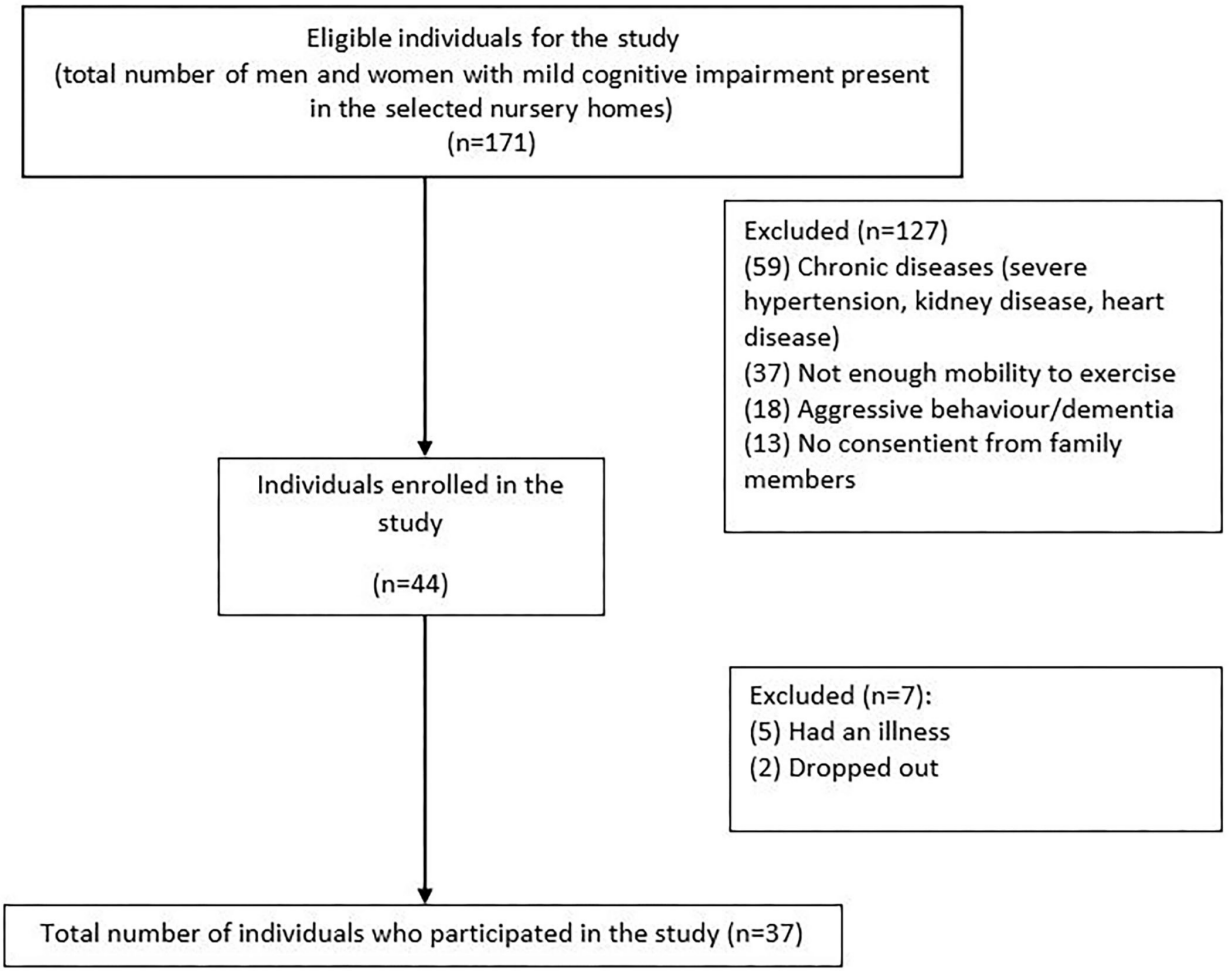
Flowchart with the inclusion and exclusion criteria of the participants.

Thirty-seven individuals aged between 64 and 97 years (*x* = 82.6; SD = 6.8; 73% female, 27% male) and living in nursing homes were included in this study. They were divided into two groups: an experimental group (EG, *n* = 25), which is submitted to 24 weeks of multicomponent exercise workout combined with cognitive stimulation (dual task), and a control group (CG, *n* = 12). The participants were recruited in five different nursing homes located in Beira Interior, Portugal, that allowed participation in the study. Inclusion criteria were mild cognitive impairment and age above 60 years. Exclusion criteria were as follows: clinical diagnosis of advanced dementia syndrome, uncontrolled hypertension (BP > 160/90 mmHg), frequent hypoglycemia, severe congestive heart, acute myocardial infarction in the last year, severe anemia (HB < 8 g dl^−1^), severe respiratory illnesses, severe osteoporosis, sensory deficit (vision/hearing) that makes it impossible to collaborate in the physical exercise program, and severe psychiatric disorders (Figure 1).

Prior to inclusion in the study, all candidates were carefully selected by a psychologist and a neurologist who performed cognitive assessments according to the standardized Mini-Mental State Examination (MMSE), and those with a diagnosis of MCI were selected.

#### Test procedures

All measurements were performed in five different nursing homes. Tests were supervised and applied to both groups before and after the application of the exercise program (baseline and post-training) by the same researchers. Each subject was familiarized with the procedures and aims of the study and gave their written informed consent. All the experimental procedures were approved according to the Declaration of Helsinki (UNESCO. Universal Declaration on Bioethics and Human Rights 2006) and were carried out with the approval of the Ethics Committee of the University of Beira Interior (reference code No. CE-UBI-PJ-2019-021).

#### Anthropometric measurement

Height (cm) was measured with a stadiometer (Cabral, model 14) with a scale of 0.10 cm, and total body weight (kg) was measured to the nearest 0.1 kg on a digital scale (Tanita, type BF511). Subjects were measured wearing shorts and t-shirts (shoes and socks were removed).

#### Physical fitness assessment

Physical fitness was assessed according to Rikli and Jones battery procedures (Rikli and Jones, 1999). Participants performed six tests: 2.44 m up-and-go test (to assess agility and dynamic balance), stand-up test (to measure lower body strength), 6-min walk test (6MWT, to assess aerobic endurance), arm curls (to measure upper body strength), sitting and reaching, and reach behind the back (to assess upper body flexibility).

#### Measurement of cognitive function

Cognitive performance was assessed by a psychologist using the MMSE questionnaire (Opasso et al., 2016), which was translated and validated for the Portuguese population (Guerreiro and Bras, 2015). This instrument consists of 11 items in a total of five domains: orientation scale (time and place), memory (recording and recall), attention/counting skill (numbering), language skill (remembering names, 3-stage order, copying, and repetition), and comprehension/judgment. A total score categorizes the individual on a scale of cognitive function ranging from 0 to 30. MMSE normative cut-off values for the Portuguese population are 22 (for 0–2 years of literacy), 24 (for 3–6 years of literacy), and 27 (for more than 6 years of literacy) (Morgado et al., 2009).

#### Blood sample collection

A venous blood sample (4 ml) was taken from each subject, between 8:30 and 10:00 a.m. in fasting conditions, and collected in ethylenediaminetetraacetic acid (EDTA) test tubes to prevent coagulation. The collected samples were then subjected to centrifugation at 3,000 rpm for 10 min, and the separated plasma was stored in Eppendorf tubes at −80°C for future analysis. The time between the last exercise session and blood sampling was between 48 and 72 h after exercise.

#### Total protein determination

Total protein concentration in plasma was spectrophotometrically estimated according to the Biuret method using serum albumin as standard (Gornall et al., 1949).

#### Lipid peroxidation

Non-specific lipid peroxidation levels in plasma were measured by determining the levels of lipid peroxides as the amount of thiobarbituric acid reactive substances (TBARS) formed, according to Wills (Gower and Wills, 1987) with some modifications. Plasma samples of 100 μl were taken and mixed with 200 μl of trichloroacetic acid (10%) and centrifuged at ~15,000 × g for 1 min. Then, 200 μl of supernatant was taken and mixed with 200 μl of thiobarbituric acid (TBA) reagent (1% thiobarbituric acid). The mixture was heated at 80–90°C for 10 min and cooled down at room temperature for 20 min. Lipid peroxidation was estimated by the appearance of malondialdehyde (MDA) which was quantified spectrophotometrically by reading the absorbance at 535 nm. The amount of MDA formed was calculated using a molar extinction coefficient (*ε*) of 1.56 × 10^5^ M^−1^ cm^−1^, and the results were expressed as MDA concentration (nmol mg^−1^ of protein).

#### Total antioxidant capacity

The total antioxidant capacity (TAC) in plasma was determined using the 2,2′-azino-bis(3-ethylbenzothiazoline-6-sulfonic acid) (ABTS) radical-scavenging activity measured by a previously reported procedure (Özgen et al., 2006) with slight modifications. A solution was prepared with ABTS^•+^ (7 mM) and potassium persulfate (140 mM) in 5 ml of distilled water. The solution was held/kept/stored at room temperature, in the dark, for 12–16 h before use. The ABTS^•+^ solution was diluted in acetate buffer (100 mM, pH 4.5), in order to obtain an absorbance of 0.7 at 734 nm. Fresh ABTS^•+^ solution was prepared for each analysis. To obtain Trolox equivalent, a standard solution was prepared at 0 (control), 1.25, 2.50, 5.00, 7.50, 10.00, 15.00, and 20.00 μM. To measure the antioxidant capacity of the samples, three different sample volumes were used. The antioxidant capacity of the samples was expressed in terms of the Trolox equivalent activity.

#### Brain-derived neurotrophic factor

Plasma BDNF concentrations were analyzed by enzyme immunoassay using ELISA kits by Millipore (Temecula, CA, USA) according to the manufacturer's description and the protocol used by a previous study (Rojas Vega et al., 2006). The intensity of light was detected by a photometer (Microplate reader, ThermoFisher, New York, USA) with a 450 nm filter.

#### Intervention: Physical exercise training and cognitive stimulation (dual task)

Considering literature recommendations (Karssemeijer et al., 2017; Bruderer-Hofstetter et al., 2018; Gheysen et al., 2018; Zhang et al., 2019; Gavelin et al., 2021), a multicomponent exercise intervention was implemented, where the cognitive stimulation was incorporated into the sessions and simultaneously combined with resistance or aerobic training.

Regarding the type of cognitive stimulation for people with MCI, a previous report (Woods et al., 2012) suggests that they should be offered a range of enjoyable activities providing general stimulation for thinking, concentration, and memory usually in a social setting, such as a small group, involving a wide range of activities that aim to stimulate thinking and memory, including discussion of past and present events and topics of interest, word games, puzzles, music, and practical activities like baking or indoor gardening (Woods et al., 2012).

Taking into account these recommendations together with the type of equipment available for this intervention, the cognitive stimulation comprehended exercises based on word games, puzzles, mathematical calculations, forward and backward counting, computer exercises (Cogweb – www.cogweb.pt and brain on track – www.brainontrack.com), exergames (Blaze pod – www.blazepode.com), and games on a balance platform (Physio Sensing – www.physiosensing.net).

Cogweb and Brain on Track, being cognitive stimulation platforms, were combined with physical exercise, and the users were cycling while solving the exercises. The Blaze Pod and Physio Sensing are platforms that work on physical abilities, such as speed, balance, and coordination, and these were combined with cognitive stimulation with counting, naming, and calculating.

This stimulation was implemented in two sets of exercises: (1) repetitive: we kept some exercises to promote evolution and apply the use of acquired skills, and (2) alternated: in each session, we introduced different cognitive exercises, to maintain the innovative effect and motivation.

The cognitive stimulation exercises were progressively inserted as the individuals performed the motor tasks with gradual degrees of difficulty, and the exercises contemplated cognitive functions, such as attention, memory, calculation, language, and executive functions. Stretching and relaxation for 5–10 min were included at the end of the exercise session.

Concerning the type of exercise, functional training was chosen due to the characteristics of the participants (frailty). Subjects followed physical training for 24 weeks, with two sessions per week, 45–50 min per session, on non-consecutive days. Each session was divided into three components: 20–25 min of aerobic exercise(s) plus cognitive stimulation, 10–15 min of strength exercise(s) plus cognitive stimulation, and 5–10 min of stretching and cool-down. Aerobic exercises included walking and walking on circuits with functional tasks and pedaling. The intensity of the aerobic component was moderate to low, considering the individuals' physical conditions. No perceived exertion scales or cardio frequencies were used; however, visual indicators were used as external signs of fatigue, such as respiratory rate, speech fluency, and blushing (Solomon, 2006; Riley et al., 2018). The strength exercises were mostly calisthenics for the lower and upper limbs, and for arm flexion, dumbbells between 1 and 3 kg were used. The intensity of the strength component varied between 65 and 75% of the maximum of one repetition per minute (1RM), three sets of 8–12 repetitions in the first 4 weeks, and three sets of 12–16 repetitions in the following weeks.

To calculate 1RM by the indirect method, a repetitions test was used given the frailty of the study population.

#### Statistical procedures

All statistical analyses were performed using the Statistical Package for Social Sciences (SPSS) software version 21. The alpha level was set to 0.05, and data results were shown as mean ± SD. The normality of distribution was checked with the Shapiro–Wilk test, and the homogeneity of variance was tested by Levene's statistics. The effect of physical exercise on physical fitness variables, total MMSE score, lipid profile, BDNF, TAC, and MDA was assayed using a general linear model (GLM) repeated measures. For the non-parametric variables (chair sit-and-reach, 6-min walking, MMSE subscales, and total cholesterol), a Mann–Whitney test was performed for intra-group comparisons.

## Results

The respective mean values and standard deviation (SD) of age and education of the sample are described in Table 1.

**Table 1:** Mean ± standard deviation values of age and education.

**Variables**	**CG (*N* = 12)**	**EG (*N* = 25)**
Age (years)	81.8 ± 8.9	83.2 ± 5.5
**Education**
Can neither read nor write	7	8
1–11 years of schooling	5	16
Higher education	0	1

The variance in the anthropometric and functional variables along the 24 weeks of intervention are described in Table 2.

**Table 2:** Mean ± standard deviation values of weight, body mass index (BMI), and functional variables, before and after intervention of exercise program.

**Variables**	**CG (*****N*** = **12)**		**EG (*****N*** = **25)**	
	**Baseline**	**Post**	**Baseline**	**Post**
Weight (Kg)	71.9 ± 13.5	72.6 ± 4.13	69.7 ± 14.1	69.9 ± 2.8
BMI (Kg/m^2^)	28.1 ± 4.9	28.3 ± 5.1	28.6 ± 4.6	28.5 ± 4.7
Chair stand (no. of stands)	6.3 ± 5.0	5.3 ± 1.8	6.8 ± 5.6	8.2 ± 1.1^#^
Arm Curls (n° of reps)	18.6 ± 4.8	17.0 ± 4.8	16.5 ± 4.5	21.1 ± 3.8^#^
Chair sit-&-Reach (cm +/–)	(–)4.7 ± 8.7	(–) 6.4 ± 8.7^*^	(–) 3.1 ± 8.3	0.4 ± 6.4^*^
8-Ft Up-&-Go (s)	16.8 ± 14.2	18.9 ± 15.2	21.1 ± 11.7	17.2 ± 10.8^#^
Back scratch (cm +/–)	(–)30.1 ± 5.6	(–)34.9 ± 5.5	(–)37.2 ± 18.1	(–)31.7 ± 17.9 ^#^
Six min walking (m)	145.3 ± 140.1	117.3 ± 126.0^*^	183.7 ± 192.8	204.3 ± 195.7
Feet together (s)	9.3 ± 1.6	9.3 ± 1.6	10.0 ± 0.0	9.8 ± 0.8
Semi Tandem (s)	6.8 ± 3.9	4.7 ± 3.3^*^	8.8 ± 3.1	8.8 ± 3.1
Full Tandem (s)	3.8 ± 4.1	1.9 ± 3.2^*^	6.2 ± 4.1	7.0 ± 4.1
SPBT	1.5 ± 1.0	0.9 ± 0.5^*^	2.3 ± 0.7	2.4 ± 0.9

# Between groups significant variation between baseline and post-intervention moment.

* Intragroup significant difference between baseline and post-intervention moment.

BMI, *body mass index;* CG, control group; EG, experimental group; SPBT, short performance battery test.

From baseline to week 24, GLM-repeated measures revealed that no body composition changes were observed in both groups, but significant variations in strength parameters were observed in both groups, in upper limbs (arm curl, *p* = 0.000), lower limbs (chair stand, *p* = 0.003), agility and dynamic balance (8 feet up-and-go; *p* = 0.002), and upper limb flexibility (back scratch; *p* = 0.002).

The nonparametric test(s) revealed that lower limb flexibility (chair sit-and-reach) decreased in the CG (*p* = 0.007) and increased in the EG groups (*p* = 0.007). Considering the remaining parameters, no significant changes were observed in the EG, while the CG showed a decreased performance in the 6-min walking test (*p* = 0.032), semi-tandem test (*p* = 0.004), full tandem (*p* = 0.017), and SPBT (*p* = 0.020).

**Figure 2:**
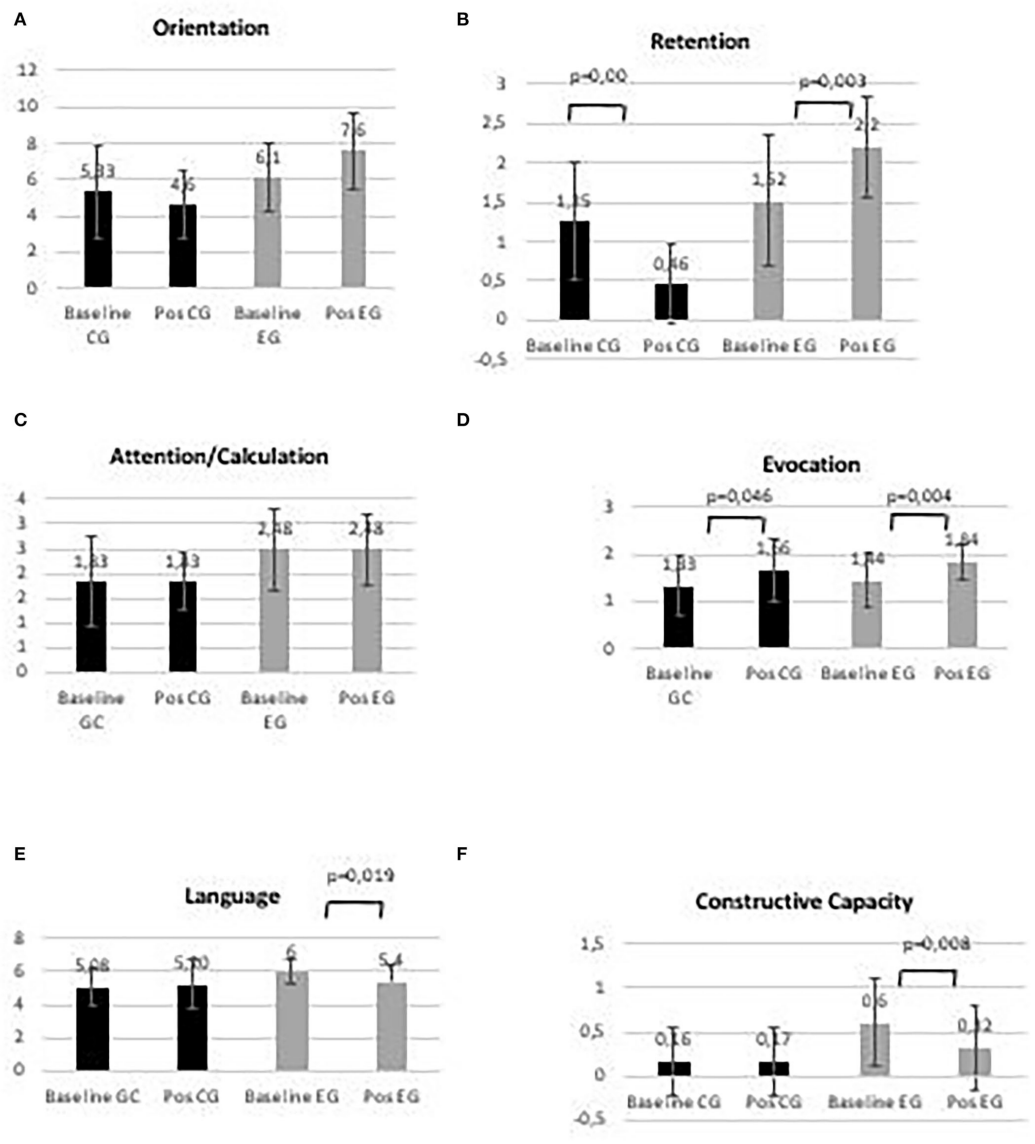
Control group (CG) and experimental group (EG), **(A)** Orientation; **(B)** Retention; **(C)** Attention/Calculation; **(D)** Evocation; **(E)** Language, and **(F)** Constructive capacity, in baseline and post-intervention period.

Cognitive function (MMSE) was significantly different between groups at baseline (CG = 15.0 ± 4.4; EG = 13.1 ± 3.9) and after 24 weeks of intervention (CG = 18.2 ± 2.6; EG = 19.8 ± 3.2), with multicomponent exercise exerting a significant effect on the mental function of both groups (*p* = 0.000). MMSE subscale variations with the intervention can be observed in Figure 2.

No significant changes in orientation and attention/calculation between baseline and post-intervention period were observed in both groups. Significant differences between baseline and post-intervention periods were observed in both groups for retention (*p* = 0.000 and *p* = 0.003, for CG and EG, respectively). An increase in evocation between baseline and post-intervention period was observed in both groups (*p* = 0.046 and *p* = 0.004, for CG and EG, respectively). Language and constructive capacity decreased in the EG, between baseline and post-intervention period (*p* = 0.019 and *p* = 0.008, respectively).

**Figure 3:**
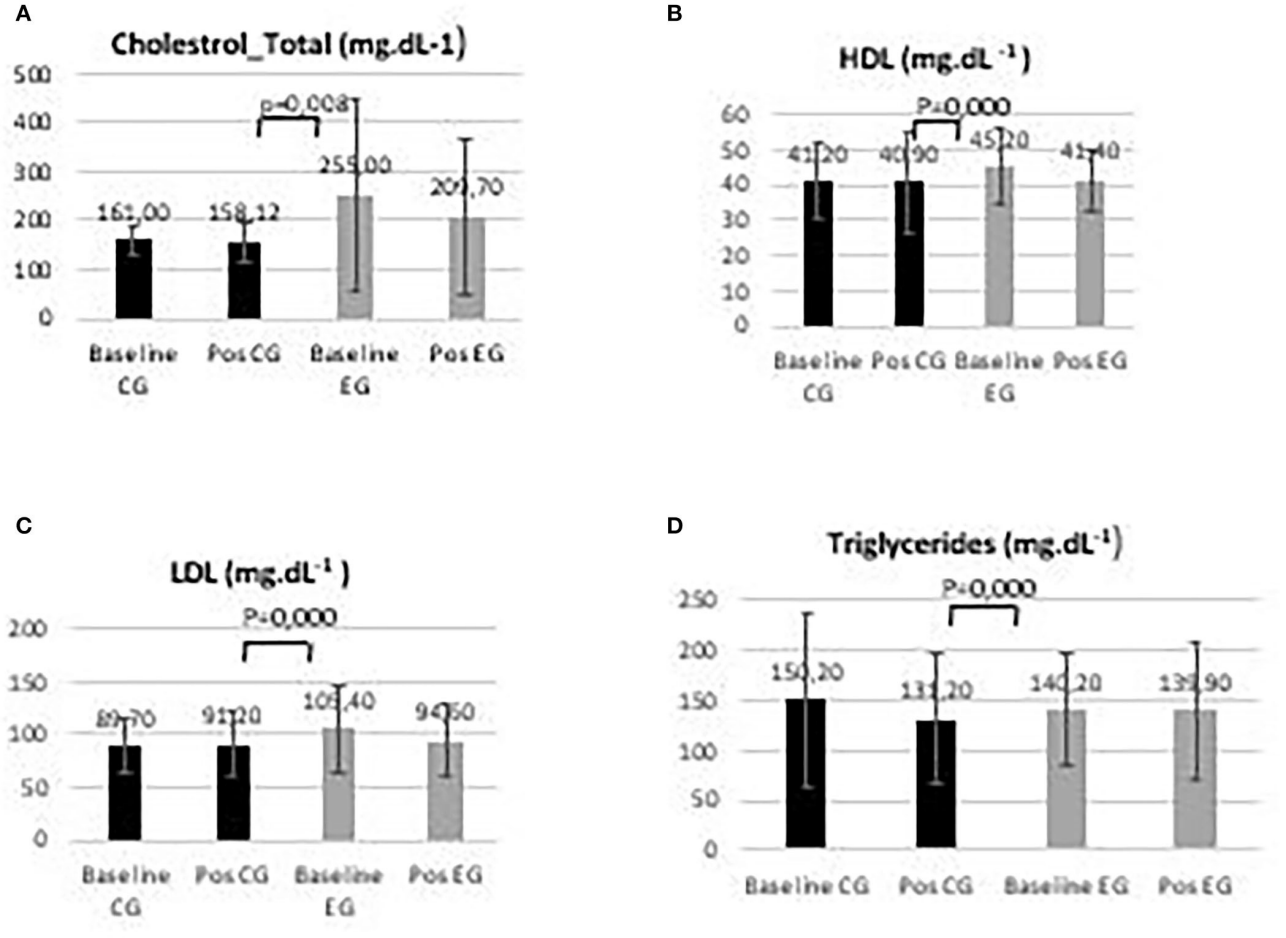
Control group (CG) and experimental group (EG) plasmatic lipid profile **(A)** total cholesterol; **(B)** high-density lipoprotein, HDL; **(C)** low-density lipoprotein, LDL; and **(D)** triglycerides in baseline and post-intervention period.

Plasmatic lipid profile variations during the intervention period are presented in Figure 3.

Regarding lipid profile variables, significant differences in the levels of total cholesterol (*p* = 0.008), HDL (*p* = 0.000), LDL (*p* = 0.000), and triglycerides (*p* = 0.000) were observed in the groups between baseline and post-intervention.

**Figure 4:**
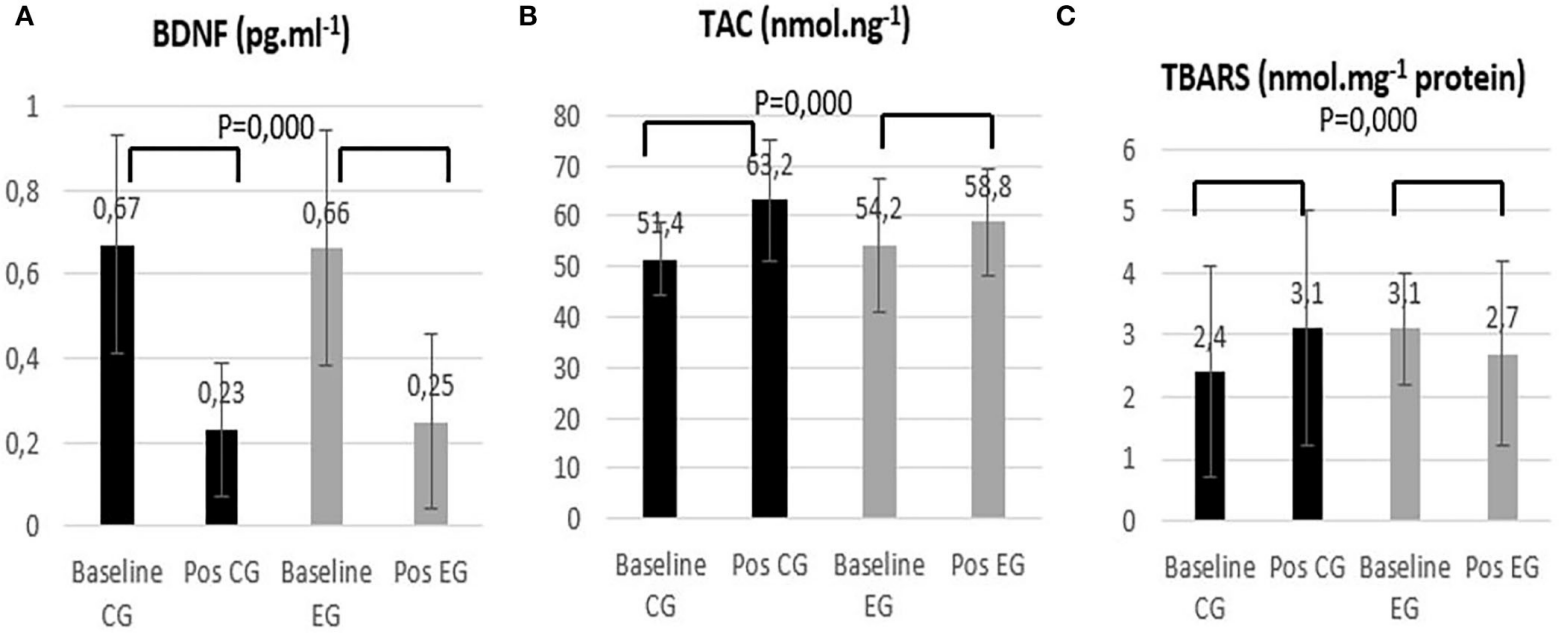
Control group (CG) and experimental group (EG) **(A)** Brain-derived neurotrophic factor (BDNF); **(B)** Total antioxidant capacity (TAC); and **(C)** Thiobarbituric acid reactive substances (TBARS) in baseline and post-intervention period.

The variations in the levels of plasma biochemical parameters like BDNF, lipid peroxidation (TBARs), and total antioxidant capacity (TAC) during the intervention period are presented in Figure 4.

Considering the plasmatic concentration of BDNF, there was a significant decrease in both groups between the baseline and post-intervention (*p* = 0.000). The total antioxidant capacity increased in both groups (*p* = 0.000). With respect to TBARS, it is possible to notice that while the CG group showed increased lipid peroxidation (*p* = 0.000) damage, the EG group showed decreased damage (*p* = 0.000).

## Discussion

This study aimed to analyze the effect of a multicomponent physical exercise program with stimulation on fitness, cognitive function, plasmatic lipidic profile, oxidative stress, and BDNF of elderlies with MCI. Our results revealed a significantly crucial positive improvement in fitness in the EG group, while the CG group showed a decrease in almost all the fitness variables. These results enhance the importance of regular exercise in delaying the aging process. This is even more important considering the fragility of the elderly living in nursing homes. The values obtained describe a sample with a BMI above the recommended values and values in the physical fitness parameters lower than those indicated for healthy elderly people of the same age (Rikli and Jones, 2013; São Romão Preto et al., 2015; Sampaio et al., 2019). Nevertheless, these results are corroborated by other studies that are also conducted on elderlies living in nursing homes (São Romão Preto et al., 2015). Indeed, although the aging process induces physical and physiological impairments, such as decreased muscular strength, power, and mobility in all elderly people, they seem to be more pronounced in those who spend long periods of time physically inactive (e.g., sitting and watching TV) (Oesen et al., 2015; Sampaio et al., 2019). This is a very common reality in nursing homes (Oesen et al., 2015; Scarabottolo et al., 2017; Sampaio et al., 2019), which may explain the low levels of functional fitness in our sample. It should also be added that several studies report an inverse association between the level of daily physical activity and cognitive function in the elderly (Brett et al., 2016; Marmeleira et al., 2017), which is aggravated by the aging process (Chougnet et al., 2015; Scarabottolo et al., 2017; Grimmer et al., 2019). Regarding the CG, a significant loss in all parameters of functional fitness was observed after 6 months without intervention. On the other hand, in the EG, it was possible to observe a significant improvement in several parameters, and in the remaining ones, there was no decrease in the performance of the elderly, which clearly indicates the benefits of physical exercise in delaying the loss of functionality associated with age. Although these results are corroborated by other studies (Lobo et al., 2011; Pedrero-Chamizo et al., 2012; Scarabottolo et al., 2017), it is important to emphasize the characteristics of the implemented exercise program. In this case, strength exercises were privileged, due to the sample frailty, enhancing balance and reducing the risk of falling (Schwenk et al., 2010; Coelho et al., 2013; Shin and An, 2014; Borges et al., 2015; Sobol et al., 2016), combined with functional circuits and dual-tasks situations (the primary task was motor and the secondary task was cognitive) (He et al., 2018), to stimulate cognitive ability.

Regarding cognitive function, the values reveal the existence of a cognitive deficit in both groups, which was more pronounced in the EG group. Contrary to our expectations, both groups showed an increase in total cognitive ability at the post-test moment compared to the baseline situation. Nevertheless, this improvement was more expressive in the experimental group. One would expect maintenance or decrease in the cognitive function in the CG group, as a result of the aging process, as has been described in other studies (Gregory et al., 2017; Rezola-Pardo et al., 2019; Zhang et al., 2019). However, some variables were not controlled during the study and could explain the enhancement of cognitive function, particularly in the non-intervened group. In fact, other strategies have also been recommended in the treatment of MCI, such as the use of antioxidants, medication, and lifestyle changes (DeCarli, 2003; Odawara, 2012; Eshkoor et al., 2015).

Considering the six MMSE subscales, the results also raise some inconsistencies. Despite the intense and diverse cognitive stimulation, the variable that changed in a distinct and significant way was retention, which decreased in the CG group and increased in the EG group. Regarding the remaining variables, the results were not very consistent, as there were improvements/losses or maintenance in both groups. These results may be justified, in part, by uncontrolled factors independent of the program performed, as already mentioned above, but may also express limitations of the instrument used in the assessment of cognitive function. The MMSE has several advantages, such as its translation and validation into more than 50 languages (Carnero-Pardo, 2014) which makes it possible to find comparative norms in the most varied circumstances, evaluates different cognitive domains, is easy to apply and interpret, and establishes cut-off points for cognitive deficits (Guerreiro and Bras, 2015) according to education level. Thus, this instrument seems, from the outset, to fit the population studied, as approximately 40% of the sample is illiterate (57% have between 1 and 12 years of schooling and 2% have higher education). The sample's education status made it impossible to use other validated instruments, such as the Montreal Cognitive Assessment (MOCA) (Hobson, 2015). On the other hand, the MMSE has several limitations, including the fact that it was not created specifically for the detection of dementia. This explains why most of its score is due to orientation (10 points) and language (eight points), and only three of its 30 points assess memory, a cognitive domain that is primarily and early affected in MCI (Carnero-Pardo, 2014) and which is the most affected by our multicomponent exercise program, also reported in another study (Grober and Sliwinski, 1991). Executive functions are also underrepresented in the MMSE, making it an instrument with little sensitivity to frontal dysfunctions (Carnero-Pardo, 2014). The MMSE contains several items that do not provide much discriminatory ability on the whole, especially in MCI or dementia.

Regarding the lipid profile, significant variations were observed between groups. The values of the sample at baseline show normal or close to normal values, which, given its high body mass index, suggests taking medication and/or taking care of food and other healthy lifestyle habits (Barre et al., 2011; Barnard et al., 2014). The exercise intervention may have helped to accentuate some of these improvements observed in the EG.

The variations in BDNF were very similar in both groups, decreasing between the two evaluation moments. These results somewhat contradict our expectations, as BDNF is an important neurotrophin involved in neuroplasticity and cognitive function, so an increase in BDNF in the EG would be expected. However, despite the results of studies carried out in animals, particularly mice, they are quite consistent in showing an increase in BDNF with different modes of exercise(s), whereas in humans, the results are very controversial, with some reporting an increase (Zoladz et al., 2008; Erickson et al., 2011; Voss et al., 2013), some reporting no change (Goekint et al., 2010; Church et al., 2016), and some reporting a decrease (De la Rosa et al., 2019). BDNF is known to induce rapid excitation and transmitter release, and to facilitate long-term potentiation through TrkB receptors and MAP kinase activation (Kafitz et al., 1999). MAP kinase activation is involved in mitochondria biogenesis, a cell-signaling pathway also stimulated by exercise, particularly intense exercise (Radak and Taylor, 2019). So, we would expect an increase in energy capacity production resulting both from BDNF and exercise, which could explain the increase in cognitive function.

However, in our study, BDNF decreased in both groups, suggesting that the age-dependent effect is more relevant than exercise. Our results also failed to prove the importance of BDNF in the cognitive function of the elderly with MCI, and once the CG revealed the same cognitive and BDNF variation as EG.

Regarding the oxidative stress parameters, we found a very beneficial effect of multicomponent exercise, as it induced a significant decrease in oxidative damage (TBARs) in the EG, also corroborated in the literature in studies with independent elderly people (Soares et al., 2015; Mota et al., 2019). This result can be explained through the multicomponent exercise effect on the activation of redox-sensitive signaling pathways, such as NF-B, heat-shock transcriptional factor 1 (HSF-1), and P53 pathways, as well as mitogen-activated protein kinase (MAPK) and an increase in antioxidant capacity (Ji, 2006), also found in this study. However, results describing the multicomponent exercise-related changes in oxidative stress of elderlies living in nursing homes were difficult to identify. Regarding the CG, an increase in oxidative damage was observed, despite the increase in the antioxidant capacity, which suggests that a more intense generation of reactive oxygen species occurred in this group. This variation in the oxidative stress parameters in the CG also indicates the influence of possible uncontrolled variables in the study (food, supplementation, and medication) that might have influenced TAC.

## Conclusion

The results suggest that a multicomponent exercise training program (aerobic and strength exercises combined with cognitive stimulation) in institutionalized elderly with MCI is effective for improving physical fitness, memory, and reducing damage induced by oxidative stress. The combined exercise program may be a method to mitigate the aging processes associated with oxidative stress. Additional studies are necessary to clarify the role of regular exercise in BDNF and its effect on cognitive function. It is suggested that future studies on elderlies with MCI should take food and medication into consideration.

### Study limitations

This study has some limitations: (1) the sample size (37 participants, predominantly female), (2) the fact that overall, participants were classified as overweight and obese, (3) the age (participants were very old), and (4) several participants were polymedicated, and this aspect was not taken into account.

Another limitation concerns the lack of control of several variations, such as diet, use of antioxidant supplementation, medication, and lifestyle changes. These variables may explain the improvement in cognitive function, particularly in the non-intervention group.

It is also important to refer to as a limitation the presence of some differences in some baseline measurements between the groups.

The use of the TBARS technique is another limitation that should be considered.

## Data availability statement

The original contributions presented in the study are included in the article/supplementary material, further inquiries can be directed to the corresponding author.

## Ethics statement

The studies involving human participants were reviewed and approved by the Ethics Committee of the University of Beira Interior (reference code No. CE-UBI-PJ-2019−021). The patients/participants provided their written informed consent to participate in this study.

## Author contributions

Conceptualization: CR and MM. Methodology, formal analysis, investigation, data curation, writing-original draft preparation, and software: CR. Validation: CR, DE, and MM. Resources, project adminitraton, and visualization: DE. Writing—review and editing and supervision: MM. Funding acquisition: DE and MM. All authors have read and agreed to the published version of the manuscript.

## Funding

This work was supported by CIDESD (NORTE-01-0145-FEDER-000083).

## Conflict of interest

The authors declare that the research was conducted in the absence of any commercial or financial relationships that could be construed as a potential conflict of interest.

## Publisher's note

All claims expressed in this article are solely those of the authors and do not necessarily represent those of their affiliated organizations, or those of the publisher, the editors and the reviewers. Any product that may be evaluated in this article, or claim that may be made by its manufacturer, is not guaranteed or endorsed by the publisher.
